# Enhancing Preoperative Orthopaedic Communication: A Comparative Analysis of Large Language Model- and Clinician-Generated Clinic Letters

**DOI:** 10.7759/cureus.101413

**Published:** 2026-01-13

**Authors:** Wilfred C Saunders, Alexander C Glendenning, Charles Gamble, Richard Roberts

**Affiliations:** 1 Emergency Medicine, Wrexham Maelor Hospital, Wrexham, GBR; 2 Trauma and Orthopaedics, Morriston Hospital, Swansea Bay University Health Board, Swansea, GBR; 3 Trauma and Orthopaedics, Wrexham Maelor Hospital, Wrexham, GBR; 4 General Surgery, Wrexham Maelor Hospital, Wrexham, GBR

**Keywords:** artificial intelligence in medicine, clinic letters, large language model, orthopaedic clinic, patient communication, readability analysis, surgical consent

## Abstract

Background

Clear, effective communication is fundamental to orthopaedic practice, particularly when securing informed consent. Escalating NHS workforce and time constraints necessitate tools that streamline, yet enhance, patient‑clinician dialogue. By analysing understandability, readability, and complication profile inclusion, this study aims to determine the feasibility of large language model (LLM)‑assisted correspondence to support equitable, patient‑centred consent and decision‑making.

Methods

Six frequently performed orthopaedic operations were chosen. Standardised, clinic‑friendly prompts were fed to four LLMs: OpenAI o1, DeepSeek, Gemini, and Copilot, each producing two letters per procedure. An identical prompt was provided to two clinicians to produce letters for the same operation, serving as a human benchmark. Understandability (Patient Education Materials Assessment Tool for Printable Materials (PEMAT-P)), readability (Flesch-Kincaid readability tests, Gunning Fog Index, and Simple Measure of Gobbledygook (SMOG) indices), and gold-standard complication inclusion were recorded.

Results

PEMAT-P understandability scores for each LLM were as follows: OpenAI o1 0.72 (±0.07), DeepSeek 0.81 (±0.09), Copilot 0.81 (±0.08), Gemini 0.83 (±0.05). Human letters scored 0.72 (±0.03). All LLMs produced text at a seventh-eighth grade level; Flesch‑Kincaid 6.850-8.517, markedly simpler than human letters (10.6 ± 0.94). OpenAI o1’s outputs were easiest to read according to the Gunning-Fog and SMOG scales (8.8833 ± 0.5702 and 9.9833 ± 0.4569), whereas clinician letters were harder (14.1333 ± 1.1 and 13.3333 ± 0.55).  OpenAI o1 achieved the greatest complication profile compliance (0.923 ± 0.104, P < 0.001), followed by Gemini (0.860 ± 0.079).

Conclusion

LLMs can outperform traditional clinician correspondence in readability and understandability, while simultaneously incorporating gold‑standard complication profiles into clinic letters. Embedding optimised, LLM workflows within outpatient practice could markedly reduce administrative burden, minimise transcription delays, and empower patients to make better‑informed, shared decisions. Future research must refine LLM search capability, evaluate cost‑effectiveness, ensure ethical and medico‑legal oversight, integrate outputs with electronic health records, and establish rigorously validated pathways for safe clinical deployment.

## Introduction

Clinicians in the United Kingdom face ever-growing pressures within the NHS, with particular emphasis and pressure from the media and general public, due to increasing waiting list times. Evidence suggests that within the United Kingdom, there is a 30.1% increase in the number of patients on elective orthopaedic waiting lists over a 12-month period [1]. Furthermore, clinic data demonstrated an 18.9% rise in clinic volumes between 2018 and 2022, with elective clinics running late 25% of the time [2]. Although innovations such as electronic medical documentation have reduced errors and shortened waiting times in several outpatient settings, these gains unfold against an expanding backlog that still demands urgent, system-wide attention [3]. 

Current techniques to reduce waiting times for orthopaedic clinics include physiotherapy-led clinics, pre-clinic X-rays, and triaging services [2]. Horler et al. found through use of surveys in outpatient musculoskeletal departments that both patients and healthcare professionals considered clinic letters to be a crucial part of patient care, but time constraints were identified as the main barrier to achieving this [4].

However, with the introduction of further technological advancements into the clinic environment, concern exists regarding the loss of the shared decision-making process [5]. Furthermore, following the Supreme Court case of Montgomery vs Lanarkshire, increased importance is placed on providing patients with all information about the procedure, including risks most relevant to them [6]. This highlights the importance of clearly understandable communication between clinicians and patients. Research has demonstrated that patients have improved comprehension and satisfaction with both written and verbal information; however, increasing time pressures on clinicians may lead to deficiencies in patient care and difficulties providing this information [7]. Recent evidence now suggests that artificial intelligence (AI), such as large language models (LLMs), can not only improve clinician-patient interaction but also improve the consenting process [8,9].

AI is an area of growing interest and use in medicine. It refers to the use of computer systems to simulate human intelligence through the analysis and interpretation of information [10]. LLMs are a subset of AI, and include various models such as OpenAI o1 (OpenAI, San Francisco, California, United States), Google Bard (Google LLC, Menlo Park, California, United States), and DeepSeek (Hangzhou DeepSeek Artificial Intelligence Basic Technology Research Co., Ltd., Hangzhou, Zhejiang, China). These work by synthesising data from multiple sources to summarise information on a topic and produce high-quality and understandable text by learning the relationships between input and output sequences [11]. Through this method, LLMs can generate texts quickly, which can be adapted and tailored by clinicians, presenting an innovative opportunity to enhance healthcare communication and administrative efficiency. Studies have shown that LLMs are effective medical documentation tools that can significantly improve the efficiency and accuracy of clinical notes [11]. More recent evidence also suggests that LLMs may be able to generate clinic letters that incorporate up-to-date complication profiles [12]; however, a paucity of evidence exists when comparing this to human dictation.

This study explores how effectively LLMs can synthesise information from relevant sources to generate clear and comprehensive preoperative clinic letters for common elective orthopaedic operations versus human-generated letters with the same prerequisite information. We assess the ability of the LLMs to incorporate gold-standard complication profiles. This will help to assess the proficiency of different LLMs in generating effective clinic letters for elective orthopaedic operations and will add to the existing literature on the potential uses and benefits of utilising AI in medicine.

This article was previously presented as an abstract at the British Orthopaedic Association Annual Congress on September 16, 2025, and as a poster presentation at the Royal College of Surgeons Manchester Foundation Trainee Surgical Society (MFTSS) Future of Surgery Conference on July 20, 2025.

## Materials and methods

This study was conducted at the Wrexham Maelor Hospital, Wrexham, United Kingdom. Six commonly performed elective orthopaedic operations were selected: total hip replacement, total knee replacement, arthroscopic anterior cruciate ligament reconstruction, arthroscopic meniscus repair, arthroscopic rotator cuff repair, and carpal tunnel release [13]. Complication profiles for each operation were sourced from the American Academy of Orthopaedic Surgeons (AAOS) website [14]. The AAOS was selected over the British Orthopaedic Association Standards for Trauma and Orthopaedics (BOASTs) [15] as the AAOS had freely available guidelines for each of the six operations. This allowed direct comparison of adherence to the complication profile for each letter.

**Figure 1:**
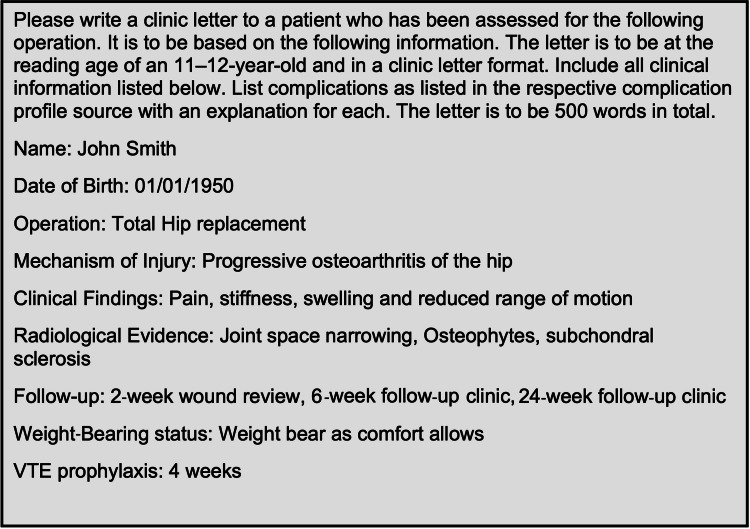
Prompt used to create a clinic letter for a patient being assessed for total hip replacement

To generate the letters, a distinct prompt was created (Figure 1). The prompt was designed in combination with senior orthopaedic surgeons to ensure the appropriate information was included in each letter. This included the following sections: Proposed operation, Mechanism of Injury, Clinical findings, Radiological evidence, Follow-up, Weight-bearing status, Venous thromboembolism prophylaxis, and Complications. Statistics were deliberately not included within the letters as evidence has demonstrated that, without a clinician's explanation, these numbers can be misinterpreted and therefore patients can inappropriately place excess weight on certain risks [16]. American guidelines suggest that patient-information should be at the reading age of an 11-12-year-old [17]. Therefore, the prompts included instructions to produce letters at this reading level.

Four LLMs were selected (OpenAI o1 [18], DeepSeek [19], Gemini [20], and Copilot (Microsoft Corporation, Redmond, Washington, United States) [21]). The prompt was input into each LLM between January 22 and February 16, 2025, to generate the clinic letters. In addition to this, two clinicians dictated sample clinic letters for each operation. This enabled comparison between clinician-dictated letters and the proposed innovative LLM-based technique.

The letters were analysed according to three domains, and statistical analyses were performed for each domain using Bonferroni-adjusted P values. Firstly, each letter was assessed using the Patient Education Materials Assessment Tool (PEMAT-P) to evaluate the understandability [22]. This system analyses each letter according to 19 different topics, assigning a score of 1 if the letter agrees with the topic, and 0 if not. An overall PEMAT-P score of 1 is the highest possible, and 0 is the lowest. Only the understandability score of the PEMAT-P was calculated, as the actionability section was not appropriate in this scenario. The PEMAT-P understandability tool allows clinicians to reliably assess patient education material in terms of health literacy demands [22]. Three assessors scored each letter and were blinded to the source of the letter.

**Figure 2:**
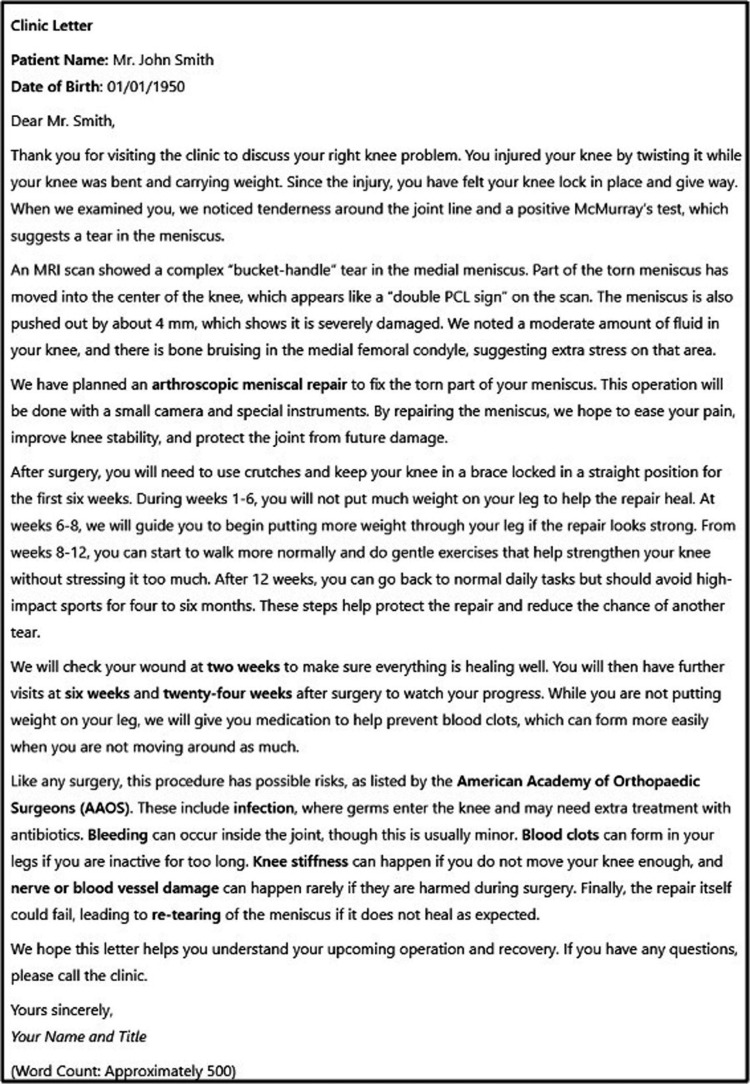
A sample of an OpenAI-o1-generated clinic letter for a patient being assessed for arthroscopic meniscal repair

Secondly, the online tool Readable [23] was used to determine readability as used by other studies [24]. The Flesch-Kincaid [25,26], Gunning Fog [27,28], and Simple Measure of Gobbledygook (SMOG) [29] scoring systems were used (Figure 2). The Flesch-Kincaid and Gunning-Fog systems determine the reading age of the text through analysing the word and sentence length. The SMOG index analyses 30 sentences in the text to calculate the reading level, and is a system widely used in healthcare. All three tools generate scores which are equivalent to United States school grades; for example, a score of 6 represents 6th grade in the United States, or 11 to 12 years old.

The readability formulae for the calculation of (i) Flesch-Kincaid readability score, (ii) Gunning-Fog readability score, and (iii) SMOG reading score have been generated as:

(i) $0.30 \left( \frac{Total ~Words}{Total ~Sentences} \right) + 11.8 \left( \frac{Total ~Syllables}{Total ~Words} \right)-15.59$,

(ii) $0.4\left\{ \left(\frac{Total ~Words}{Total ~Sentences}\right)+100\left( \frac{Complex~Words}{Total~Words} \right) \right\}$, and

(iiii) $1.0430\sqrt{Number ~of ~Polysyllables\times \frac{30}{Number ~of ~Sentences}} +3.1291$, respectively.

Finally, adherence to the complication profile was assessed. Each risk identified in the AAOS guidelines was collected, and then compared to the complications identified in each letter, generating a percentage adherence. As the clinicians dictating the letters were previously aware of the AAOS complications for each operation, clinician letters were not scored according to adherence to the complication profile.

## Results

A total of 60 clinic letters were produced: 48 were generated by the four different LLMs (Figure 2), with a further 12 human letters. Each of these letters adhered to the prescribed length and formatting based on the prompt instructions.

Understandability

PEMAT-P understandability scores for each LLM were as follows: OpenAI o1 0.72 (±0.07), DeepSeek 0.81 (±0.09), Copilot 0.81 (±0.08), Gemini 0.83 (±0.05). Human letters scored 0.72 (±0.03) (Table 1). When compared to human letters, Gemini, Copilot, and DeepSeek were all significantly more understandable (P<0.05) (Table 2).

**Table 1:** Mean PEMAT-P understandability scores for large language model outputs and clinician-dictated letters Data presented as mean±SD PEMAT-P: Patient Education Materials Assessment Tool for Printable Materials

	Copilot	DeepSeek	Gemini 2.0	OpenAI o1	Human
PEMAT-P	0.81 ± 0.08	0.81 ± 0.09	0.83 ± 0.05	0.72 ± 0.07	0.72 ± 0.03

**Table 2:** Mean differences between PEMAT-P understandability scores of clinician-dictated letters and LLM outputs with Bonferroni-adjusted P values PEMAT-P: Patient Education Materials Assessment Tool for Printable Materials; LLM: large language model

Human		LLMs	Mean Difference	P Value
Clinician Letters	Copilot (Microsoft Corporation, Redmond, Washington, United States)		-0.0833	<0.05
	DeepSeek (Hangzhou DeepSeek Artificial Intelligence Basic Technology Research Co., Ltd., Hangzhou, Zhejiang, China)		-0.0857	<0.05
	Gemini 2.0 (Google LLC, Menlo Park, California, United States)		-0.1042	<0.05
	OpenAI o1 (OpenAI, San Francisco, California, United States)		-0.0046	1

Readability

Table 3 shows the Flesch-Kincaid, Gunning-Fog, and SMOG readability scores for the LLMs and human letters, also detailing the respective ages for each grade. Human letters were hardest to read for each scoring index: 10.5750 (Age 15-16 years), 14.1333 (Age 17-20 years), and 13.3333 (Age 17-20 years) for Flesch-Kincaid, Gunning-Fog, and SMOG readability scores, respectively. OpenAI o1 produced letters with the lowest reading grades, ranging from Flesch-Kincaid grade 6.8500 (Age 11-12 years), Gunning-Fog grade 8.8833 (Age 13-14 years), and SMOG grade 9.9833 (Age 14-15 years). Gemini, DeepSeek, and Copilot scored reading grades of 8.2250-11.5000 (Age 13-17 years), 8.4917-11.4583 (Age 13-17 years), and 8.5167-11.5833 (Age 13-17 years), respectively. As demonstrated in Table 4, LLM letters were significantly more readable (p<0.001) than human letters.

**Table 3:** Descriptive statistics for readability (grade level) of letter by LLMs and clinicians across readability formulae. SMOG: Simple Measure of Gobbledygook

Readability Scoring Scales	Letter sources	Reading Age – Grade	Age (Years)
Flesch-Kincaid	Copilot (Microsoft Corporation, Redmond, Washington, United States)	8.5167	13-14
	DeepSeek (Hangzhou DeepSeek Artificial Intelligence Basic Technology Research Co., Ltd., Hangzhou, Zhejiang, China)	8.4917	13-14
	Gemini 2.0 (Google LLC, Menlo Park, California, United States)	8.2250	13-14
	OpenAI o1 (OpenAI, San Francisco, California, United States)	6.8500	11-12
	Human	10.5750	15-16
Gunning-Fog	Copilot	10.4912	15-16
	DeepSeek	10.5167	15-16
	Gemini 2.0	10.6750	15-16
	OpenAI o1	8.8833	13-14
	Human	14.1333	17-20
SMOG	Copilot	11.5833	16-17
	DeepSeek	11.4583	16-17
	Gemini 2.0	11.5000	16-17
	OpenAI o1	9.9833	14-15
	Human	13.3333	17-20

**Table 4:** Mean differences between readability scores of clinician-dictated letters and LLM outputs with Bonferroni-adjusted P values. LLM: large language model; SMOG: Simple Measure of Gobbledygook

Readability Scoring Scales	Letter sources		Mean Differences	P Value
Flesch-Kincaid	Human	Copilot (Microsoft Corporation, Redmond, Washington, United States)	2.0583	<0.001
		DeepSeek (Hangzhou DeepSeek Artificial Intelligence Basic Technology Research Co., Ltd., Hangzhou, Zhejiang, China)	2.0833	<0.001
		Gemini 2.0 (Google LLC, Menlo Park, California, United States)	2.3500	<0.001
		OpenAI o1 (OpenAI, San Francisco, California, United States)	3.7250	<0.001
Gunning-Fog	Human	Copilot	3.6417	<0.001
		DeepSeek	3.6167	<0.001
		Gemini 2.0	3.4583	<0.001
		OpenAI o1	5.2500	<0.001
SMOG	Human	Copilot	1.7500	<0.001
		DeepSeek	1.8750	<0.001
		Gemini 2.0	1.8333	<0.001
		OpenAI o1	3.3500	<0.001

Complication profile adherence

**Figure 3:**
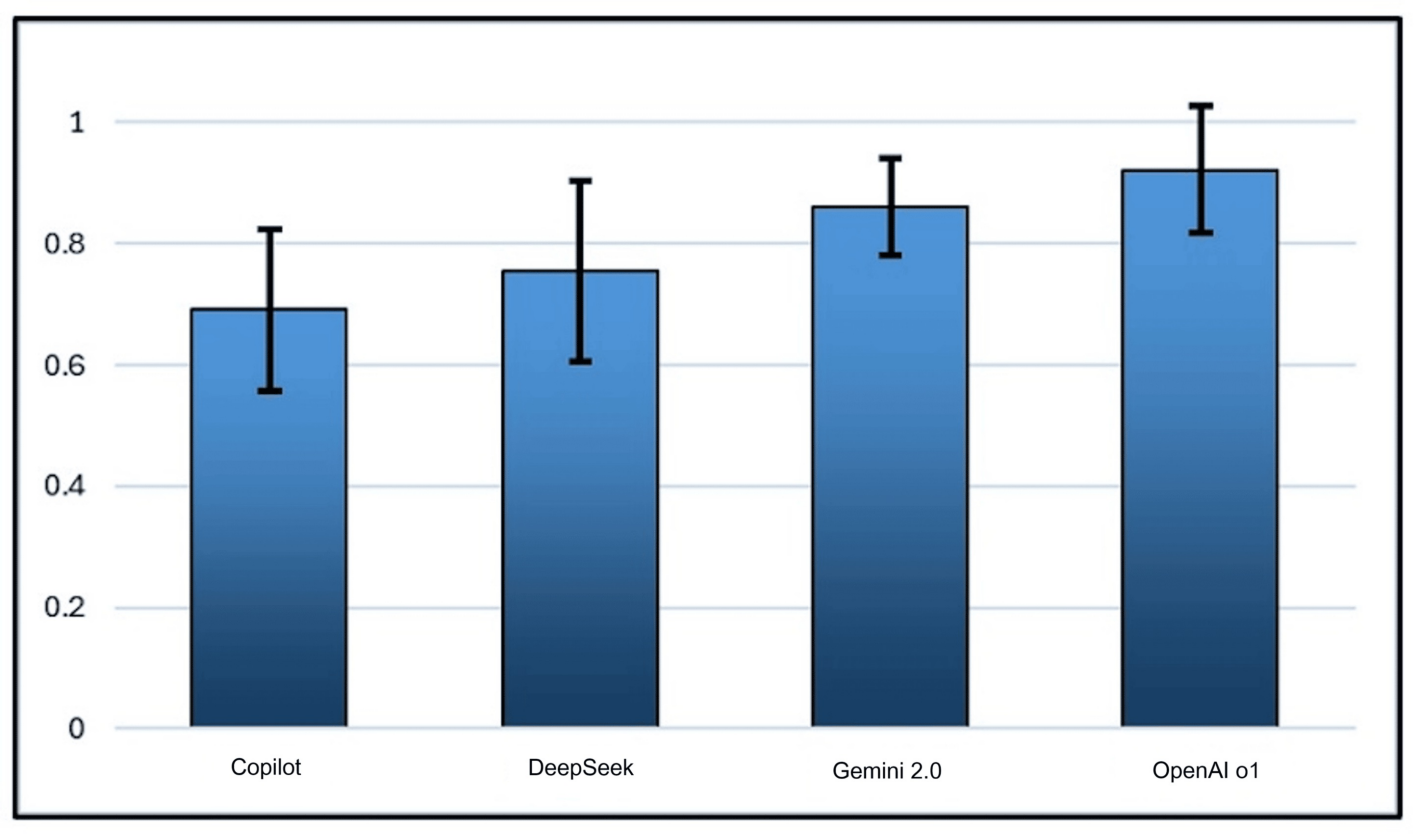
Mean proportion of guideline-recommended postoperative-complication instructions correctly included in clinic letters generated by four LLMs LLM: large language model

OpenAI o1 demonstrated the highest complication profile adherence (0.923 ± 0.104), followed by Gemini (0.860 ± 0.079), DeepSeek (0.756 ± 0.149), and then Copilot (0.691 ± 0.132) (Figure 3). OpenAI o1 was significantly more adherent to the complication profile when compared to DeepSeek and Copilot (p<0.05 and p<0.001), and Gemini was more adherent than Copilot (p<0.05) (Table 5). OpenAI o1 and Gemini complication profile adherence scores were significantly better than Copilot (P<0.001 and P<0.05, respectively).

**Table 5:** Mean differences between complication profile adherence between LLM outputs Bonferroni-adjusted P values. LLM: large language model

LLM		Mean Difference	P Value
Copilot	DeepSeek (Hangzhou DeepSeek Artificial Intelligence Basic Technology Research Co., Ltd., Hangzhou, Zhejiang, China)	-0.0650	0.546
	Gemini 2.0 (Google LLC, Menlo Park, California, United States)	-0.1700	<0.05
	OpenAI o1 (OpenAI, San Francisco, California, United States)	-0.2317	<0.001
DeepSeek	Copilot (Microsoft Corporation, Redmond, Washington, United States)	0.0650	0.546
	Gemini 2.0	-0.1050	0.152
	OpenAI o1	-0.1667	<0.05
Gemini 2.0	Copilot	0.1700	<0.05
	DeepSeek	0.1050	0.152
	OpenAI o1	-0.0617	0.589
ChatGPT-O1 O1	Copilot	0.2317	<0.001
	DeepSeek	0.1667	<0.05
	Gemini 2.0	0.0617	0.589

## Discussion

Our proof-of-concept study shows that contemporary LLMs can generate elective orthopaedic clinic letters that are more readable, more understandable, and crucially capture a broader range of procedure-specific risks than letters dictated by experienced clinicians working under usual time constraints.

The PEMAT-P understandability score outlines that a score of greater than 0.7 or 70% indicates that patients are more likely to find understandable and actionable patient education materials [22]. A study investigating the understandability of online material for anterior cruciate ligament surgery found that only 12.82% of the resources scored PEMAT-P scores of 70% or higher [30]. Additionally, research has demonstrated that the average PEMAT-P score of online shoulder arthroscopy information was 61.33 [31]. Our study demonstrates that all generated letters achieved a PEMAT-P score of greater than 0.7, with Gemini, DeepSeek, and Copilot all producing significantly more understandable letters than traditional clinician-dictated letters. Therefore, LLMs can produce more understandable information than humans and online material, allowing for improved health literacy and better patient-led autonomous decision-making.

Health Education England suggests patient information be written at the level of 11-year-olds [32], while the American Medical Association recommends an age range of 11-12 years [17]; however, research has shown that current orthopaedic patient education material on the AAOS is significantly greater than this recommendation, with average Flesch-Kincaid grade scores of 8.9 [33]. A study assessing the readability of elective orthopaedic patient information found that only 13.7% was set at the recommended reading age [34]. In our study, all LLM-generated letters scored lower Flesch-Kincaid grades and were significantly lower in score than the human letters, indicating that LLMs can produce effective and more readable patient material than is currently available. The capacity to understand and interpret information is paramount in informed consent. This study demonstrates that LLMs, particularly OpenAI o1, can produce significantly more readable letters than humans, which are also easier to understand than the currently available orthopaedic patient information and therefore can potentially act as an aid in the process of obtaining consent. 

The results of our study illustrate high adherence to the complication profile, demonstrating that they can successfully generate patient information using predefined complication profiles. The standardisation provided by LLMs can potentially reduce clinical oversight risks, promoting safer, more thorough patient consent. However, it is important to note that none of the LLMs demonstrated 100% adherence for every single operation. This highlights a crucial limitation of using LLMs. Montgomery v Lanarkshire established a legal precedent ensuring that patients receive comprehensive risk information tailored to their specific concerns [6]. This study proposes an integrated approach between LLM and the clinician, where the clinician can tailor the information in the prompt. The combination of clinician experience and LLM efficiency promotes efficient and specific doctor-patient communication.

LLMs are capable of synthesising and processing large quantities of data with extreme efficiency. However, their reliability is critically dependent on the quality of the data. Previous studies have shown concerns regarding the risk of LLMs making clinical mistakes [35]. LLMs are vulnerable to publicity bias and inherited errors, which can lead to skewed outputs [36]. To mitigate these risks, prompts must be carefully constructed to ensure high-quality outputs. For example, in this study, key clinical information was included, as well as pre-defining the source of the complications using a nationally recognised guideline to ensure a patient-relevant and accurate letter is produced, which still mitigates delays in documentation production. 

It is important to note that there are key ethical and financial concerns surrounding artificial intelligence. Healthcare documentation contains sensitive patient-identifiable data. Patient confidentiality is essential to maintaining trust between patient and doctor. It is therefore vital to ensure that LLM systems comply with legal regulations such as the General Data Protection Regulation [37].

Our study outlines an innovative method of using LLMs and expert clinician feedback to generate understandable, accurate, and patient-specific clinical letters for common orthopaedic operations, building on previous research on LLM use in clinical documentation. AI remains a relatively novel technology and is continuously evolving. Its use in healthcare is developing rapidly, and this study shows promising evidence for LLM implementation whilst providing scope for further research, such as its use in other specialties, discharge letters, and patient information.

## Conclusions

When combined with clinician input and feedback, LLMs generate high-quality clinical letters for common orthopaedic operations that are clear, readable, and comprehensive in communicating risks and complications. In addition, it can be used to enhance the consent process and set a new standard in patient-centred clinical communication, aligning with clinical best practice and medico-legal requirements. This highlights an innovative suggestion to streamline documentation time and clerical workload, reduce clinician time pressures, and improve outpatient department efficiency. Further research should examine patient and clinician responses to standard dictated clinical letters versus LLM-generated letters, as well as analysing real-world clinical outcomes prior to full integration of LLMs into routine orthopaedic practice.
